# Sustained Mood Improvement with Laughing Gas Exposure (SMILE): Study protocol for a randomized placebo-controlled pilot trial of nitrous oxide for treatment-resistant depression

**DOI:** 10.1371/journal.pone.0297330

**Published:** 2024-01-19

**Authors:** Karim S. Ladha, Jiwon Lee, Gabriella F. Mattina, Janneth Pazmino-Canizares, Duminda N. Wijeysundera, Fatemeh Gholamali Nezhad, Kaylyssa Philip, Vanessa K. Tassone, Fathima Adamsahib, Venkat Bhat, on behalf of the SMILE Study Investigators

**Affiliations:** 1 Department of Anesthesiology and Pain Medicine, University of Toronto, Toronto, Ontario, Canada; 2 Department of Anesthesia, St. Michael’s Hospital, Toronto, Ontario, Canada; 3 Temerty Faculty of Medicine, University of Toronto, Toronto, Ontario, Canada; 4 Interventional Psychiatry Program, St. Michael’s Hospital, Toronto, Ontario, Canada; 5 Institute of Medical Science, University of Toronto, Toronto, Ontario, Canada; 6 Neuroscience Research Program, St. Michael’s Hospital, Toronto, Ontario, Canada; 7 Department of Psychiatry, Temerty Faculty of Medicine, University of Toronto, Toronto, Ontario, Canada; University of Connecticut Health Center: UConn Health, UNITED STATES

## Abstract

**Background:**

Nitrous oxide has shown potentially as an efficacious intervention for treatment-resistant depression, yet there remains insufficient evidence pertaining to repeated administration of nitrous oxide over time and active placebo-controlled studies with optimal blinding. Thus, we aim to examine the feasibility and preliminary efficacy of a six-week follow up study examining the effects of a 4 week course of weekly administered nitrous oxide as compared to the active placebo, midazolam.

**Methods:**

In this randomized, active placebo-controlled, pilot trial, 40 participants with treatment-resistant depression will receive either inhaled nitrous oxide (1 hour at 50% concentration) plus intravenous saline (100mL) or inhaled oxygen (1 hour at 50% concentration) plus intravenous midazolam (0.02 mg/kg in 100mL, up to 2mg) once per week, for 4 consecutive weeks. Participants will be followed up for 6 weeks starting from the first treatment visit. Primary feasibility outcomes include recruitment rate, withdrawal rate, adherence, missing data, and adverse events. The primary exploratory clinical outcome is change in Montgomery-Åsberg Depression Rating Scale (MADRS) score at day 42 of the study. Other exploratory clinical outcomes include remission (defined as MADRS score <10), response (defined as ≥ 50% reduction in MADRS score), and adverse side effects.

**Discussion:**

This pilot study will provide valuable information regarding the feasibility and preliminary efficacy of repeated nitrous oxide administration over time for treatment-resistant depression. If feasible, this study will inform the design of a future definitive trial of nitrous oxide as an efficacious and fast-acting treatment for treatment-resistant depression.

**Trial registration:**

ClinicalTrials.gov NCT04957368. Registered on July 12, 2021.

## Introduction

Major depressive disorder (MDD) represents a highly prevalent and debilitating psychiatric illness, ranking among the top leading causes of disability worldwide [1]. While numerous antidepressant medications are available, around 30% of patients with MDD are treatment-resistant, which is typically defined as persisting symptoms despite trials of two antidepressants [2,3]. Patients with treatment-resistant depression (TRD) are much less likely to respond to additional trials of antidepressants and, in turn, face poorer prognosis [4]. Even when efficacious, antidepressants often require several weeks to take clinical effect [5]. Further, antidepressants present with a range of side effects [6,7], leading to significant patient burden. The perceived burden of antidepressants persists throughout the course of treatment and associate with poorer treatment outcomes [7], posing further challenges to treating MDD. Given the high rates of TRD along with the shortcomings of current antidepressant treatments, there is a major need for novel therapies with different mechanisms of action for TRD that are tolerable, efficacious, and fast-acting.

A candidate pharmacological agent for treatment of TRD is nitrous oxide, an inhalational anesthetic also known as ‘laughing gas’. Nitrous oxide has been suggested to target symptoms of MDD through its non-competitive inhibition of N-methyl-D-aspartate (NMDA) receptors, which in turn, have been implicated in the pathophysiology of MDD [8]. Specifically, postmortem brains of MDD patients demonstrate abnormal expression of NMDA receptor subtypes and intracellular proteins involved in NMDA receptor signaling, while inhibition of NMDA receptors have shown to induce symptoms of depression in preclinical models. Further, in line with the role of NMDA receptors in MDD, the NMDA receptor antagonist ketamine currently holds FDA approval for use in TRD [9]. Ketamine, however, has concerning side-effects such as psychotomimetic reactions unlike nitrous oxide, which has established clinical safety [10]. Additionally, the low cost of nitrous oxide along with its already widespread use across dental and medical procedures [11] renders nitrous oxide further appealing as an alternative treatment for TRD.

Currently, several studies have examined nitrous oxide treatment in the context of psychiatric disorders [12], including randomized controlled trials (RCTs) in TRD demonstrating rapid antidepressant effects of nitrous oxide [13–17]. Indeed, reductions in symptoms of depression have been observed as early as 2 hours post-treatment and sustained for 24 hours [14–16], with a subset of studies demonstrating effects at one or two weeks following nitrous oxide administration [14,15,17]. However, this line of evidence has been based on work that lacks adequate blinding of participants. Specifically, the forementioned studies selected standard placebo treatments instead of an active placebo such as midazolam, which may achieve better treatment blinding given that both nitrous oxide and midazolam exert psychoactive effects [18]. Accordingly, midazolam has been employed as an active placebo for a prior study examining the effects of nitrous oxide on mood in treatment-resistant bipolar depression [18]. Midazolam has also been widely used as an active placebo in studies testing ketamine in TRD [15], further supporting the use of midazolam as a more appropriate placebo for nitrous oxide trials in TRD. In addition to the lack of optimal blinding, current studies investigating nitrous oxide in TRD [13–17] have not conducted follow up past two weeks, and have only examined a single administration of nitrous oxide, rather than repeated administrations over time. While a recent RCT that included both patients with TRD and non-TRD suggests that repeated administrations of nitrous oxide over four weeks may produce cumulative amelioration of depressive symptoms [19], this study also lacked adequate blinding; and it is unclear whether these results can be translated to patients with TRD specifically. Thus, we aim to conduct a pilot randomized trial in patients with TRD that involves repeated once-weekly nitrous oxide administration for four weeks, use of an active placebo (*i*.*e*., midazolam), and follow-up across six weeks.

### Objectives

The primary objective of this pilot study is to assess the feasibility of conducting a double-blind, randomized, active placebo-controlled trial testing the effects of repeated nitrous oxide administration on depression symptoms in patients with TRD. The secondary objective is to examine whether repeated nitrous oxide treatment can reduce symptoms of depression at day 42 of the study as compared to midazolam.

## Methods

### Study design

**Fig 1:**
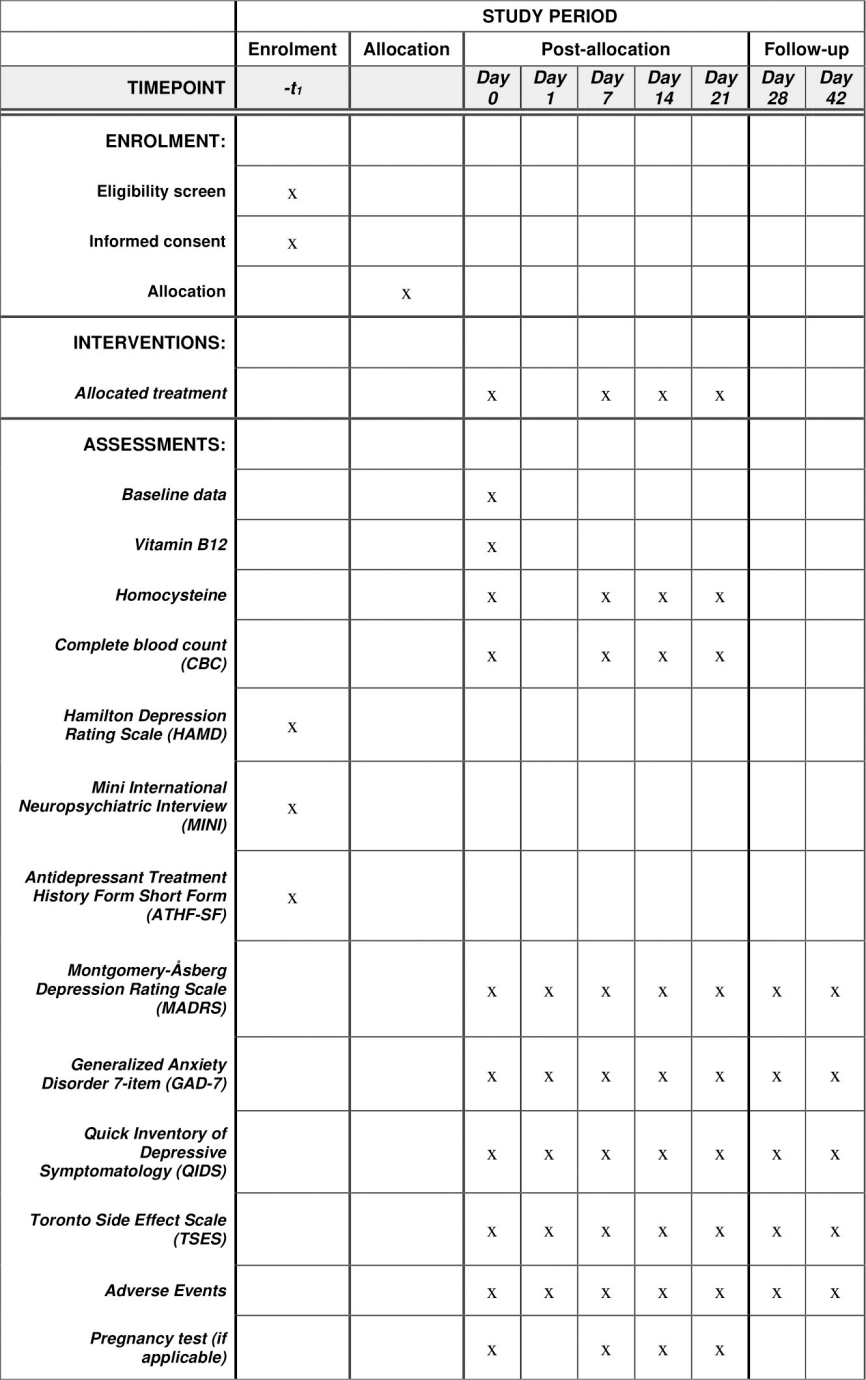
Schedule of enrolment, interventions, and assessments.

The Sustained Mood Improvement with Laughing Gas Exposure (SMILE) trial is a pilot, double-blind, randomized, active placebo-controlled trial with two parallel arms. The participants are randomized in a 1:1 ratio to either the treatment arm (*i*.*e*., nitrous oxide) or active placebo arm (*i*.*e*., midazolam). The schedule of enrolment, interventions, assessments, and visits for participants is outlined in Fig 1. The Standard Protocol Items: Recommendations for Interventional Trials (SPIRIT) checklist is available in S1 File. The full protocol (version 1.1, dated 20 January 2022) is available in S2 File.

### Study setting

This is a single center trial conducted at St. Michael’s Hospital in Canada. Patients will be recruited from the Centre for Depression and Suicide Studies at the hospital.

### Sample size

Due to the pilot nature of this study, the anticipated effect size is unknown, and a formal sample size cannot be calculated. We plan to recruit forty participants. If the study protocol is found to be feasible, the effect size in this pilot study (point estimates and confidence limits) will inform the sample size estimates for the future definitive trial.

### Participant eligibility criteria

Participants must meet all of the following inclusion criteria:

1. 18 to 65 years of age
2. Meet Diagnostic and Statistical Manual for Mental Disorders (DSM-5) criteria for MDD
3. Current major depressive episode as confirmed by the Mini International Neuropsychiatric Interview (MINI) [20] for DSM-5
4. Experiencing moderate to severe depressive episode, as defined by the Hamilton Depression Rating Scale (HAMD) [21] > 17
5. Failure of two trials of antidepressant therapy of adequate dose and duration (*i*.*e*., at or above the minimum oral dose for at least 4 weeks as defined by the Antidepressant Treatment History Form Short Form (ATHF-SF)), during the current depressive episode
6. For women of childbearing potential, use of highly effective or double-barrier methods of contraception. Abstinence is acceptable if it is the preferred and usual lifestyle of the female participant
7. Capacity to provide informed consent.

Participants who meet any of the following criteria will be excluded:

1. Acute suicidality defined as score ≥3 on HAMD item 3
2. Major depressive episode in people with bipolar disorder
3. Current substance abuse or dependence and/or history of alcohol abuse or dependence within the past year
4. Dementia
5. Current or lifetime history of schizophrenia or schizoaffective disorder
6. Current history of dissociative disorders
7. Known history of hypersensitivity or allergy to nitrous oxide, midazolam, or any ingredients in the study formulations
8. Contraindication to receiving nitrous oxide (*e*.*g*., any condition where air is entrapped within a body and its expansion might be dangerous such as pneumothorax, elevated intracranial pressure, air embolism, recent middle ear, vitreoretinal or bowel obstruction surgeries. Additionally, chronic cobalamin or folate deficiency, *i*.*e*., signs of anemia or neurological symptoms with plasma levels of homocysteine over 15 Umol/L and complete blood count (CBC) showing abnormal red blood cells and leukocytes)
9. Contraindication to receiving midazolam (*e*.*g*., shock, chronic heart failure, chronic obstructive pulmonary disease, closed-angle glaucoma, renal failure, patients with limited pulmonary reserve or those with severe decline of vital signs)
10. Use of centrally acting medicinal products, such as opioid agonists, (*e*.*g*., naloxone and naltrexone), morphine derivatives (*e*.*g*., oxicodon, hydrocodone, oximorphone, codeine), benzodiazepines (*e*.*g*., diazepam, clonazepam, altrazodam) and/or other central nervous system (CNS) depressants such as barbiturates (*e*.*g*., phenobarbital, pentobarbital, amobarbital) and alcohol
11. Pregnancy or breastfeeding in female participants
12. Electroconvulsive therapy within the current depressive episode
13. Receiving ketamine treatment within the current depressive episode
14. Unwilling to maintain current antidepressant regimen.

### Recruitment

All patients meeting DSM-5 criteria for MDD from the Centre for Depression and Suicide Studies at St. Michael’s hospital will be approached for recruitment. The clinic receives approximately 100 referrals per year, of which 30% are estimated to be eligible for inclusion in this study.

### Informed consent

The study coordinator will approach potential participants and provide information about the purpose of the study and the informed consent form. The participants will be provided with sufficient time to read through the informed consent form and ask questions before deciding to participate. Participants who consent to participate will be asked to sign the informed consent form.

### Intervention

Participants will be randomized to one of two arms: 1) the active treatment, inhaled nitrous oxide at an inspiratory concentration of 50% plus concurrent intravenous saline (100ml) for one hour; or 2) the placebo, inhaled 50% oxygen plus concurrent intravenous midazolam (0.02mg/kg in 100ml, up to 2 mg) for one hour. The inhalants will be delivered via a nitrous oxide delivery system. Following administration of nitrous oxide or oxygen, and once nitrous oxide flow stops, 100% oxygen will be delivered for a few minutes. Participants will receive their respective study treatment once a week for four weeks for a total of four treatments. An anesthesiologist will oversee intervention administration, monitoring, and post-treatment recovery. The anesthesiologist will ascertain adherence through administration of the intervention. Participants will be withdrawn from the study if they present with any severe adverse event or unknown allergic reaction during the first administration of nitrous oxide. Participants may also voluntarily withdraw for any reason. During the trial, participants will continue to receive regular psychiatric care and receive treatment for MDD during the study as prescribed by their primary responsible physician.

### Randomization and allocation concealment

The allocation sequence will be generated using an online random number generator in random permuted blocks (www.sealedenvelope.com) of varying sizes. The allocation sequence will be sent to and only be accessible by Research Pharmacy at St. Michael’s Hospital. Research Pharmacy will prepare the study package, which comprises either midazolam or normal saline vials contained in an obscure bag that look identical to each other.

### Blinding

The physician administering the study treatments will not be blinded in order to prepare the appropriate study package containing intravenous administration (saline or midazolam) and the corresponding gas inhalation (nitrous oxide or oxygen respectively). The administering physician will not be assessing outcomes to minimize observer bias. The participants, outcome assessors, data analysts, and clinicians outside those administering the study interventions will be blinded to allocation assignment. Outcome assessors will not be present during any of the study medication administrations. To minimize attrition after randomization, the interventional physician will open the study package once the participant enters the procedure room.

During the trial, emergency unblinding will occur only when knowledge of intervention is essential for participant care. The timing, reason, and personnel involved in the emergency unblinding will be recorded and blinding will be maintained in as many other study personnel as possible. Following study completion (*i*.*e*., completed the 6 weeks follow up), participants will be informed about their group allocation if requested, as it was deemed potentially harmful to withhold this information from patients with TRD who are continuously trying different treatment options.

### Outcomes

The primary feasibility outcomes are rates of recruitment, withdrawal, adherence, missing data, and adverse events. The primary exploratory clinical outcome is the change in the Montgomery-Åsberg Depression Rating Scale (MADRS) [22] score from baseline to six weeks post-treatment. MADRS is a 10-item scale designed to measure severity of MDD and detect changes in symptoms of depression due to treatment. Other exploratory clinical outcomes include remission (defined as MADRS score <10 [23]), response (defined as ≥ 50% reduction in MADRS score from baseline [24]), and adverse side effects from antidepressant treatment as assessed by the Toronto Side Effects Scale (TSES) [25].

### Assessments and collection of outcomes

#### Eligibility screen

After participants have provided informed consent, they will be assessed for study eligibility using HAMD, MINI for DSM-5, and ATHF-SF [26]. They will also be asked about their medical comorbidities and concomitant medications to confirm eligibility.

#### Baseline visit (day 0)

Baseline data include demographic information (*e*.*g*., age, sex, gender, height, and weight), current medications, medical comorbidities, medication history assessed using the ATHF-SH, and medical history (*i*.*e*., history of bipolar or psychosis, substance use, electroconvulsive treatment, and previous treatment with ketamine). Additionally, the severity of depressive symptoms will be assessed using MADRS and Quick Inventory of Depressive Symptomatology (QIDS) [27]. Anxiety symptoms will be assessed using General Anxiety Disorder 7-item (GAD-7) [28]. Adverse side effects from antidepressant treatment will be assessed using TSES. A blood sample will also be collected to measure CBC, Vitamin B12, and homocysteine levels. For females of childbearing potential, a urine pregnancy test will be completed to confirm that the participant is not pregnant prior to starting the study.

#### Follow-up visits (days 1, 7, 14, 21, 28, and 42)

During each follow-up visit, participants will complete the MADRS, GAD-7, QIDS, and the TSES to assess outcome measurements. Participants will also be asked about concomitant medications at each follow-up visit. Follow-ups that coincide with treatment administrations will be done in-person prior to the intervention. Otherwise, follow-ups will occur over the phone. At each treatment visit (days 0, 7, 14, and 21), participants will be asked for a blood sample to test for CBC and homocysteine levels before treatment administration. Females of childbearing potential will additionally be required to undergo a urine pregnancy test at each treatment visit to confirm that they are not pregnant prior to treatment administration.

### Data management

Study data will be collected using case report forms and entered into Microsoft Access database. Appropriate range and missing data filters will be used to maintain data quality. Data accuracy will be assessed by randomly selecting 10% of individuals who will have their data confirmed by a second reviewer.

### Statistical methods

For feasibility outcomes, the rates and percentages will be estimated by using lower one-sided 95% confidence intervals (CIs) for variables where high percentages are desirable (*i*.*e*., recruitment rate and adherence), and upper one-sided 95% CIs for variables where low percentages are desirable (*i*.*e*., withdrawal rate, missing data, and adverse events). Estimates will be compared with pre-specified targets to inform feasibility and the need for protocol modifications.

For clinical outcomes, statistics will be conducted descriptively and according to the intention-to-treat (ITT) principle. Data will be reported separately for each study arm. Continuous variables will be presented as means with standard deviations, or if the data is skewed, as medians with interquartile ranges. Categorical variables will be presented as counts and percentages. Given the pilot nature of this trial, the sample size is not designed to provide statistical power to detect clinically meaningful differences in outcomes. As such, between-group differences will be estimated using two-sided 95% CIs. The estimated effect size of the primary outcome (*i*.*e*., reduction in MADRS score at day 42) will be used to inform the sample size for the future definitive trial. No formal adjustments will be made to address missing data or multiple comparisons. There are no interim or additional analyses planned.

### Monitoring

Given that this is a feasibility trial with a small sample size, no data monitoring committee will be formed. Additionally, due to the pilot nature of the study, there will be no pre-specified independent audit.

### Adverse event reporting and harms

Adverse events will be collected at each visit using TSES and by checking participant records for any documented events. Any adverse events that occur during the study will be recorded in detail. If the participant reports several signs or symptoms representing a single syndrome or diagnosis, the diagnosis will be recorded. The investigator will assess the severity of all adverse events (mild, moderate, or severe), the seriousness (non-serious or serious), and the likelihood that they were related to the investigational medicinal product. For adverse drug reactions, the investigator will additionally assess the expectedness (expected or unexpected). Diseases, signs, symptoms, and laboratory abnormalities that pre-exist the first administration of investigational treatment will not be considered adverse events unless they increase in severity or frequency. Adverse events requiring action will be treated with recognized standards of medical care and will be followed up on until they have either resolved or stabilized.

### Confidentiality

All study data will be labelled with a unique study identification number. Only the Principal Investigator will have access to the key that links individual participants to their study identification number. Study data will be stored securely in locked cabinets and later transferred to a secure internal hospital server that is password protected. All study data and personally identifying information will only be accessible to authorized personnel on the research team.

### Ethics

This study has been approved by St. Michael’s Hospital Research Ethics Board (REB Study #21–096) and Health Canada. The study was registered on ClinicalTrials.gov (NCT04957368) on July 12, 2021. Protocol modifications will be communicated to relevant parties, including Research Ethics Board, Health Canada, and trial participants as applicable.

### Trial status and dissemination plans

The study began recruitment and data collection in August 2021 and is currently ongoing. Upon study completion, the results will be published in a peer-reviewed journal and presented at scientific conferences and meetings. The authors do not intend on using professional writers as part of the publication process.

## Discussion

Nitrous oxide is emerging as a potentially effective and fast-acting treatment to mitigate TRD [13–17]. Here, we present the protocol for a pilot, six-week, double-blind RCT comparing a 4-week regimen of weekly administered nitrous oxide to the active placebo, midazolam, in patients with TRD. The primary objective of the study is to assess feasibility, and the secondary objective is to assess the preliminary efficacy of nitrous oxide in reducing symptoms of depression 42 days following treatment initiation. Currently, few RCTs have examined nitrous oxide treatment for TRD, with studies showing ameliorative effects of a single administration of nitrous oxide on symptoms of depression 2 to 24 hours post-treatment [13–16], and in some studies 1–2 weeks post-treatment [14,15,17]. Yet, it remains unknown whether repeated treatments can produce cumulative amelioration of depressive symptoms over a follow-up period past two weeks and in comparison to an active placebo, like midazolam. This pilot study addresses an important knowledge gap regarding treatment of TRD by simultaneously investigating repeated administration of nitrous oxide, comparison with an active placebo, and a six-week follow-up. The growing investigation around nitrous oxide as a treatment of TRD may hold significant implications for mitigating the culminating time, costs, and frustrations associated with repeated failed trials of antidepressant treatments for patients with TRD [29].

While this study will help extend the growing literature of nitrous oxide for TRD, there are some limitations to be noted. Firstly, it is possible that the dose and specific type of antidepressant medication that the participants receive as part of their regular psychiatric care may change over the course of the study, which may confound the results. However, there are ethical challenges to restricting treatment changes for research purposes, especially considering that these are individuals with TRD who are continuously trying different treatment options to improve their symptoms. This renders it difficult to impose too many study exclusion criteria regarding treatment changes during the study for this patient population. As such, we will be asking patients about medication changes at each study visit. Additionally, to minimize the effects of treatment changes, participants will not be randomized until they have had 4 weeks of a stable antidepressant medication regimen. Another limitation may be the small sample size of the study; while this was chosen due to the pilot nature of this study, it precludes the ability to conduct statistical tests of differences in clinical outcomes between the groups. Nonetheless, there is a lack of studies investigating nitrous oxide in the context of repeated administration, comparison with an active placebo, and long-term follow up in TRD, and hence, this necessitates a pilot trial to be conducted prior to a definitive trial with a larger sample size. An additional limitation may be that midazolam may not achieve perfect blinding to nitrous oxide. While nitrous oxide is colorless, it has a slightly sweet smell and taste. Nitrous oxide also causes dissociative effects, unlike midazolam, that may enable participants to distinguish their assigned treatment. Further, participants with prior exposure to nitrous oxide may also be able to identify their treatment assignment due to the aforementioned properties of nitrous oxide. However, it is extremely difficult to completely blind participants to nitrous oxide, as this would require an active placebo that holds all of the properties of nitrous oxide except for its potential antidepressant effects. For this reason, midazolam was first used as an active placebo in a TRD study testing ketamine (and later used by numerous ketamine studies in depression), as midazolam exerts psychoactive effects but has been established to lack antidepressant properties [30–32]. It is also notable that in a prior study comparing nitrous oxide to a standard placebo (oxygen), 85% of participants correctly guessed their assignment [17], highlighting the need for an active placebo to avoid overestimating the treatment effects of nitrous oxide. Midazolam, while not perfect, may represent a better alternative placebo to nitrous oxide. Additionally, we exclude individuals who have indicated active recreational use of nitrous oxide to help maintain the integrity of blinding.

## Supporting information

S1 File SPIRIT checklist. (DOC)

S2 File Study protocol. (PDF)
