# Supplementary material for: Sustained Mood Improvement with Laughing Gas Exposure (SMILE): Study protocol for a randomized placebo-controlled pilot trial of nitrous oxide for treatment-resistant depression
Source: PLoS One. 2024 Jan 19;19(1):e0297330. doi: 10.1371/journal.pone.0297330 (PMC10798444; doi:10.1371/journal.pone.0297330)
Supplement: S2 File — (PDF) [file pone.0297330.s002.pdf]

Clinical Study Protocol

SMILE Trial

Sustained **M**ood **I**mprovement with **L**aughing gas **E**xposure**:** A Randomized Controlled Pilot Trial

**Investigational Products:**	*Nitrous Oxide*
**Indication:**	Adults patients suffering from Major Depressive Disorder
**Study Design:**	Study type: Interventional trial<br>Allocation: Randomized<br>Intervention model: Parallel-arm<br>Primary purpose: Feasibility
**Principal Investigator:**	Karim S. Ladha, MD MSc FRCPC<br>St. Michael's Hospital and the University of Toronto<br>[redacted]
**Co-Principal Investigators**	Venkat Bhat, MD MSc FRCPC DABPN<br>St. Michael's Hospital and the University of Toronto<br>[redacted]
**REB Study Number:**	
**Clinical Trials.Gov #**	
**Clinical Trial Phase:**	Phase IV (Feasibility trial)
**Planned Clinical Start:**	01-AUG-2021
**Planned Clinical End:**	31 AUG-2023
**Date of Protocol:**	20 JAN 22
**Version:**	1.1

1. STUDY OUTLINE

**Name of Sponsor:**<br>**Dr. Karim Ladha, St. Michael's Hospital**	
**Name of Investigational Products:**<br>*Nitrous Oxide*	**Protocol Identification Code:**<br>REB Study Number

**Title**	**S**ustained **M**ood **I**mprovement with **L**aughing gas **E**xposure**:** A Randomized Controlled Pilot Trial
**Sources of monetary or material support**	St. Michael's Hospital Medical Services Association Innovation Fund
**Brief title**	SMILE Trial
**Indication:**	Adults patients suffering from Major Depressive Disorder
**Condition(s) or focus of study**	Treatment-Resistant Depression
**Number of participants**	40
**Primary outcome**	Feasibility (recruitment rate, withdrawals, adherence and adverse events)
**Secondary outcome**	Improvement in symptoms of depression
**Study design**	Study type: Interventional trial<br>Allocation: Randomized<br>Intervention model: Parallel-arm<br>Primary purpose: Feasibility<br>Phase: Phase IV
**Masking**	Trial participants, Outcome assessors
**Eligibility criteria**	Inclusion criteria:<br>1. 18 to 65 years of age<br>2. Major depressive disorder without psychotic symptoms according to DSM-5 criteria<br>3. Hamilton Depression Rating Scale (HAMD)>17<br>4. Current major depressive episode as confirmed by the Mini International Neuropsychiatric Interview (MINI) for DSM-5<br>5. Failure of two trials of antidepressant therapy of adequate dose and duration, during the current episode<br>6. For women of childbearing potential, use of highly effective or double-barrier methods of contraception. Abstinence is

	acceptable if it is the preferred and usual lifestyle of the female participant<br>7. Capacity to provide informed consent<br>Exclusion criteria:<br>1. Acute suicidality defined as score ≥3 on HAMD item 3<br>2. Major Depressive Episode in people with Bipolar Disorder<br>3. Current substance abuse or dependence and/or history of alcohol abuse or dependence within the past year<br>4. Dementia<br>5. Current or lifetime history of schizophrenia or schizoaffective disorder<br>6. Current history of dissociative disorders<br>7. Known history of hypersensitivity or allergy to Nitrous Oxide, Midazolam or any ingredients in the study formulation<br>8. Contraindication to receiving nitrous oxide (e.g.any condition where air is entrapped within a body and it's expansion might be dangerous such as, pneumothorax, elevated intracranial pressure, air embolism, recent middle ear, vitreoretinal or bowel obstruction surgeries, etc.)<br>9. Chronic cobalamin or folate deficiency (e.g. signs of anemia or neurological symptoms, with plasma levels of homocysteine over 15 Umol/L and abnormal red blood cells and leukocytes on a complete blood count CBC)<br>10. Contraindication to receiving the placebo midazolam (e.g. shock, chronic heart failure, chronic obstructive pulmonary disease, closed-angle glaucoma, renal failure, patients with limited pulmonary reserve or those with severe depression of vital signs)<br>11. Use of centrally acting medicinal products, such as opioid agonists (e.g. naloxone and naltrexone), morphine derivatives (e.g. oxicodon, hydrocodone, oximorphone, codeine) , benzodiazepines (e.g. diazepam, clonazepam, altrazodam) and/or other central nervous system depressants such as barbiturates (e.g. phenobarbital, pentobarbital, amobarbital) and alcohol<br>12. Pregnancy or breastfeeding in female participants<br>13. Electroconvulsive therapy within the current depressive episode<br>14. Receiving ketamine treatment within the current depressive episode<br>15. Unwilling to maintain current antidepressant regimen.

**Test Products, Dose, and Mode of Administration:**	Active treatment: Nitrous oxide will be administered at an inspiratory concentration of 50% with concurrent intravenous saline (100ml) for one hour.<br>Placebo: Oxygen will be administered at 50% with intravenous midazolam (0.02mg/kg in 100ml, up to 2 mg) for one hour
**Follow-Up:**	DAYS: 1, 7, 14, 21, 28, 42

PROTOCOL SIGNATURES

Tittle: **S**ustained **M**ood **I**mprovement with **L**aughing gas **E**xposure**:** A Randomized Controlled Pilot Trial

This study is intended to be conducted in compliance with the protocol, Good Clinical Practice and applicable regulatory requirements.

Principal Investigator

Karim Ladha MSc MD FRCPC

Assistant Professor
Department of Anesthesia & Institute of Health Policy, Management and Evaluation,
University of Toronto

[redacted]

Co-Principal Investigator

Venkat Bhat MD MSc FRCPC DABPN

Assistant Professor
Department of Psychiatry University of Toronto

[redacted]

_______________________________________ _______________________________

ABBREVIATIONS

ATHF-SF	Antidepressant Treatment History Form – Short Form
ADR	Adverse Drug Reaction
AE	Adverse Event
CBC	Complete Blood Count
CNS	Central Nervous System
CRF	Case Report Form
DSM-5	Diagnostic and Statistical Manual for Mental Disorders
HAMD	Hamilton Depression Rating Scale
IMP	Investigational Medical Product
GAD-7	General Anxiety Disorder 7-item
MADRS	Montgomery-Åsberg Depression Rating Scale
MDD	Major Depressive Disorder
MINI	Mini International Neuropsychiatric Interview
NMDA	N-methyl-D-aspartate
QIDS	Quick Inventory of Depressive Symptomatology
REB	Research Ethics Board
TSES	Toronto Side Effect Scale

Table of Contents

1. STUDY OUTLINE 2
PROTOCOL SIGNATURES 5
ABBREVIATIONS 6
2.1. Background and rationale 9
2.2. Objectives 11
2.3. Trial design 11
3. METHODS 11
3.1. Study setting 11
3.2. Eligibility criteria 11
3.2.1. Inclusion criteria 11
3.2.2. Exclusion criteria 11
3.3. Withdrawal Criteria 12
3.4. Interventions 12
3.4.1. Intervention description 12
3.4.2. Concomitant medication 13
3.4.3. Concomitant care 13
3.5. Outcomes 14
3.6. Participant timeline 14
3.6.1. Study Timeline 14
3.7. Sample size 15
3.8. Recruitment 15
3.9. Allocation 15
3.10. Blinding (masking) 15
3.10.1. Blinding mechanism 15
3.10.2. Emergency unblinding 16
3.11. Data collection 16
3.11.1. Trial procedures and evaluations 16
3.11.1.1. Pre-study Screening: 16
3.11.1.2. Baseline Data: 16
3.11.1.3. Follow-up Data: 17
3.11.1.4. Retention: 17

3.12. Data management 17
3.13. Statistical methods 17
3.13.1. Outcomes 17
3.14. Data monitoring 17
3.14.1. Formal committee 17
3.14.2. Interim analysis 18
3.15. Safety/harms 18
3.15.1. Safety of the Intervention: 18
3.15.2. Adverse Event Definitions 18
3.15.3. Collection of AEs 19
3.15.4. Severity of AEs 19
3.15.5. Causality of AEs 19
3.15.6. Classification of ADRs by Expectedness 20
3.15.7. Outcome of AEs 22
3.15.8. Procedures to manage Adverse Events 22
3.15.9. Serious Adverse Events (SAEs) 22
3.15.10. SAE Reporting Timelines 23
3.16. Auditing 23
4. ETHICS AND DISSEMINATION 23
4.1. Research ethics approval 23
4.2. Protocol amendments 24
4.3. Informed consent process 24
4.4. Declaration of interests 24
4.5. Ancillary and post-trial care 24
4.6. Dissemination policy 24
4.6.1. Trial results 24
4.6.2. Authorship 24
5. STUDY ADMINISTRATION 25
5.1. Key contacts 25
5.2. Funders 25
5.3. Roles and responsibilities 25
5.3.1 Sponsor and funder 25
6. REFERENCES 26

2. INTRODUCTION

2.1. Background and rationale

Psychiatric disorders are responsible for almost one quarter of the global burden of diseases, with depression being the foremost contributor.[1] While there are numerous available antidepressants, nearly one third of participants still remain resistant to them.[2] In Ontario, this translates to 400,000 individuals who are considered "treatment-resistant" and have a low likelihood of getting better. Thus, there is a great need to discover new interventions with alternate mechanisms of action to treat this debilitating disorder.

One of the main challenges in treating depression is that disruptions brain networks underlying depression are still not fully understood. The clinical manifestations of depression are heterogenous, which likely reflects a diversity of underlying neural circuitry and neurotransmitter dysfunction. Psychiatrists currently apply a trial and error approach, switching classes of antidepressant medications in order to find the right "fit". This is highly problematic since (1) it can take weeks to determine whether a drug is effective or not, and (2) the right drug is often not identified. The low rate of efficacy and long latency period of pharmacotherapies increase the risk for suicide and other self-injurious behaviors, highlighting the need to identify antidepressants that act faster and are more effectively. New drugs are being developed and in the pipeline for approval; however, very few move beyond phase 3 trials and those that do are typically not covered by drug plans and are often prohibitively expensive.

An alternative to creating entirely novel agents is to repurpose existing drugs that have been used for decades for other indications and have established safety. This approach mitigates many of the regulatory and safety hurdles that prohibit new drugs coming to market and speeds up the implementation process. Agents that target N-methyl-D-aspartate (NMDA) and gamma-aminobutyric acid (GABA) receptors have demonstrated particular promise for treating depression.[3] Anesthetic drugs and gases routinely administered to participants undergoing surgery have been shown to rapidly work on these same receptor targets to alter consciousness. Despite the quick onset/offset of these drugs, there is evidence that brief exposures can lead to long term effects on neuronal plasticity.[4] Unlike other experimental therapies, anesthetics are already widely available with a demonstrated safety profile when administered in an appropriate manner. Thus, anesthetic agents, which have been safely given to millions of participants, may represent an exciting new class of treatments to target depression.

Nitrous oxide is an anesthetic gas that is frequently encountered in dental offices, although it is also used in a variety of settings including labor and delivery units and operating rooms. Also known as "laughing gas" due to its euphoric effects experienced by some, nitrous oxide is a colourless, non-flammable gas with a faint sweet odour and taste. It is inexpensive and is on the World Health Organization's list of essential drugs. Nitrous oxide is believed to work as an NMDA receptor antagonist, a mechanism shared with ketamine.[5] NMDA receptors are activated by glutamate, the main excitatory neurotransmitter in the brain, and play key roles in mediating structural and functional changes in the brain. It has been hypothesized that the gluatamatergic system is involved in the pathology of depression, as administration of NMDA receptor antagonists that block NMDA receptor function have antidepressant effects in preclinical animal models and humans.[6,7] Unlike ketamine, nitrous oxide is devoid of side effects such as

psychotomimetic reactions, and results from several preliminary trials show marked improvement in patients with depression.[5]

A single administration of nitrous oxide has been shown to decrease depressive symptoms sustained for 24 hours and in some participants at least a week in a proof-of-concept trial.[8] However, the trial was limited in its investigation by only studying a single administration of the gas, inadequate blinding, and a lack of long-term follow-up. In response to these promising findings, a RCT examining nitrous oxide in patients with MDD is currently underway in the United States and Australia.[9] Recently, a group from Brazil published their findings which reported a significant reduction in depressive symptoms following repeated administration of nitrous oxide across four weeks in patients with MDD.[10] However, the major limitation of these studies is that none have been designed with an adequate control to blind participants. We intend to address these shortcomings by conducting a pilot study, using repeated administrations of the gas, having an adequate control and following participants across six weeks over the course of the study.

Nitrous Oxide Information
American Hospital Formulary Service (AHFS): 28:04.16 INHALATION ANESTHETICS
Anatomical Therapeutic Chemical (ATC): N01AX13 NITROUS OXIDE
Active ingredient group (AIG) number: 0110468001
Chemical Abstracts Service (CAS) number: 10024-97-2
Nitrous oxide is a compressed gas classified as an anesthetic inhalent.

For this feasibility study, we will use Nitrous Oxide (50% mg/ml) byLINDE CANADA LIMITED
DIN 02014467

As per the Nitrous Oxide Product Monograph (Lexicomp®, 3/1/2021). Reactions to nitrous oxide are characteristic of those associated with other anesthetic inhalents, which may include nausea, vomiting and headaches. Inappropriate use can be serious, hence nitrous oxide will be administered only by medical personnel trained in the appropriate techniques and in an adequate environment in this trial.

Midazolam Information
American Hospital Formulary Service (AHFS): 28:24.08 BENZODIAZEPINES
Anatomical Therapeutic Chemical (ATC): N05CD08 MIDAZOLAM
Active ingredient group (AIG) number: 0119935002
Chemical Abstracts Service (CAS) number: 59467-70-8
Midazolam is a substance classified as a benzodiazepine.

For this feasibility study, we will use Midazolam (0.02mg/kg in 100ml, up to 2 mg) by FRESENIUS KABI CANADA LTD. DIN 02242904 (1 mg/ml)

As per the Product Monograph MIDAZOLAM INJECTION (August 4, 2016). Reactions to intravenous midazolam are characteristic of those associated with benzodiazepines. The effects of intravenous midazolam overdose can be serious, hence intravenous midazolam will be

administered only by personnel trained in general anesthesia and skilled in early detection of hypoventilation, maintenance of a patent airway and support of ventilation in this trial.

2.2. Objectives

The primary objective of this study is to enroll 40 participants with treatment-resistant depression and assess the feasibility of conducting a double-blind, randomized, placebo-controlled, parallel-arm trial to examine whether nitrous oxide can reduce symptoms of depression on Day 42 of the study.

2.3. Trial design

This study will be a double-blind, randomized, placebo-controlled trial with two parallel arms.

3. METHODS

3.1. Study setting

This will be a single center trial where interventions will be performed at St. Michael's Hospital in a monitored setting. All participants will be recruited from the Centre for Depression and Suicide Studies at St. Michael's Hospital.

3.2. Eligibility criteria

3.2.1. Inclusion criteria

1. 18 to 65 years of age
2. Meeting Diagnostic and Statistical Manual for Mental Disorders (DSM-5) criteria for Major Depressive Disorder (MDD)
3. Current major depressive episode as confirmed by the Mini International Neuropsychiatric Interview (MINI) for DSM-5
4. Experiencing moderate to severe depressive episode, as defined by the Hamilton Depression Rating Scale (HAMD)>17
5. Failure of two trials of antidepressant therapy of adequate dose and duration, during the current depressive episode
6. For women of childbearing potential, use of highly effective or double-barrier methods of contraception. Abstinence is acceptable if it is the preferred and usual lifestyle of the female participant
7. Capacity to provide informed consent.

3.2.2. Exclusion criteria

1. Acute suicidality defined as score ≥3 on HAMD item 3
2. Major Depressive Episode in people with Bipolar Disorder
3. Current substance abuse or dependence and/or history of alcohol abuse or dependence within the past year
4. Dementia
5. Current or lifetime history of schizophrenia or schizoaffective disorder
6. Current history of dissociative disorders
7. Known history of hypersensitivity or allergy to Nitrous Oxide, Midazolam or any ingredients in the study formulations

8. Contraindication to receiving nitrous oxide (e.g. any condition where air is entrapped within a body and it's expansion might be dangerous such as, pneumothorax, elevated intracranial pressure, air embolism, recent middle ear, vitreoretinal or bowel obstruction surgeries, etc.)
9. Chronic cobalamin or folate deficiency (e.g. signs of anemia or neurological symptoms, with plasma levels of homocysteine over 15 Umol/L and abnormal red blood cells and leukocytes on a complete blood count CBC)
10. Contraindication to receiving the placebo midazolam (e.g. shock, chronic heart failure, chronic obstructive pulmonary disease, closed-angle glaucoma, renal failure, patients with limited pulmonary reserve or those with severe decline of vital signs)
11. Use of centrally acting medicinal products, such as opioid agonists, (e.g. naloxone and naltrexone) morphine derivatives (e.g. oxicodon, hydrocodone, oximorphone, codeine) , benzodiazepines (e.g. diazepam, clonazepam, altrazodam) and/or other central nervous system depressants such as barbiturates (e.g. phenobarbital, pentobarbital, amobarbital) and alcohol
12. Pregnancy or breastfeeding in female participants
13. Electroconvulsive therapy within the current depressive episode
14. Receiving ketamine treatment within the current depressive episode
15. Unwilling to maintain current antidepressant regimen.

3.3. Withdrawal Criteria

Participant will be withdrawn if the participant presents with any severe adverse event or unknown allergic reaction during the first administration of nitrous oxide, or if the participant wishes to withdraw from the study for any reason.

Participant will receive the necessary treatment to alleviate any untoward event and will be monitored by the study physician until deem to be safe to be discharged. Participants will be followed up the next day to find out about their condition.

Data from a participant who has been withdrawn will be collected up to next day after the withdrawal, to ensure that the participant has no more complications.

If a participant withdraws from the study at any time, the reasons for withdrawal will be collected and documented as part of a feasibility outcome for this study.

Since this is a feasibility study and there is no sample size required to analyze any outcome of the study, participants will not be replaced or added.

3.4. Interventions

3.4.1. Intervention description

Participants will be randomized to one of two arms. They will either receive: 1) the active treatment, administration of nitrous oxide at an inspiratory concentration of 50% with concurrent intravenous saline (100ml) for one hour; or 2) the placebo, administration of 50% oxygen with intravenous midazolam (0.02mg/kg in 100ml, up to 2 mg) for one hour.

The inhalants will be delivered via the nitrous oxide delivery system (NITRONOX HOSPITAL UNIT. Porter Sentry 34 C) which is licensed under Health Canada (Lincence number 89848). The Porter Sentry system is designed to operate on medical nitrous oxide and medical oxygen from cylinders and/or medical gas piping systems and deliver a 50% oxygen and 50% nitrous oxide gas mixture to adults in a medical setting. At the end of a nitrous oxide/oxygen administration, 100% oxygen will be delivered to patients for a few minutes when the flow of

nitrous oxide is stopped. In medical settings, recovery after being given nitrous oxide is quick, with most patients fully recovering after 20-30 minutes.

Intravenous administration of saline or midazolam ensures that participants are blinded to the nature of their intervention. Midazolam is a benzodiazepine with a quick-onset of action and short elimination half-life. It will serve as an active placebo due to its similar behavioral effects as nitrous oxide (i.e. sedation and disorientation) and widespread use in ketamine trials.
After the study intervention, participants will have their vital signs and adverse events monitored for on hour. The anesthesia provider will be responsible to assess the participant before discharge. Participants will not be allowed to drive after the intervention, therefore measures will be taken to ensure the participant has an adequate plan of transportation (i.e., a companion or a suitable transportation).

Participants will receive their respective study treatment once a week for four weeks for a total of 4 treatments.

For safety reasons, the anesthesia provider administering the medication will not be blinded. However, all other clinical and study personnel will be made unaware of the treatment assignment. Several measures will be taken to ensure blinding: anesthesia providers' interactions with participants will be scripted, medical personnel involved in the procedure will be trained to avoid unblinding, and participants will be draped to limit their field of view.

3.4.2. Concomitant medication
Participants will be required to continue with their corresponding treatment for MDD as prescribed by their psychiatrists. Before the intervention, investigators will analyze all medications taken by the participants on regular bases.

Patients taking other centrally acting medicinal products, such as opioid agonists, morphine derivatives, benzodiazepines and/or other CNS depressants, will be excluded from the study, as concomitant administration of nitrous oxide may result in increased sedation, and consequently have effects on respiration, circulation and protective reflexes. Nitrous oxide inactivates vitamin B12 and potentiates the effects of methotrexate on folate metabolism, which is why we will exclude patients with folate deficiency from participating in this study and will monitor plasma homocysteine levels and complete blood count (CBC) at several time points throughout the study. Please find more information on the "Drug Interactions" section of the Nitrous Oxide Lexicomp® product monograph (see page 3).

Patients administered midazolam with concomitant use of barbiturates, alcohol, opioids or other CNS depressant agents are especially susceptible to adverse reactions and excessive and/or prolonged drug effect. As a result, patients taking these substances will be excluded from the study.

3.4.3. Concomitant care
Regular psychiatric care will be provided as per the participant's primary responsible physician without restriction.

3.5. Outcomes

The primary outcome of this pilot trial is to determine the feasibility of a large-scale multi-center trial. Feasibility outcomes will include recruitment rate, withdrawal rate, adherence, missing data, and adverse events.

The primary clinical outcome will be a continuous measure of change (from baseline to last treatment visit) in the Montgomery-Åsberg Depression Rating Scale (MADRS), a 10-item clinician rated scale designed to measure major depressive disorder severity and detect changes due to treatment.[11] Secondary outcome measures will include remission (defined as MADRS score < 10), response (defined as ≥ 50% reduction in MADRS score from baseline), and adverse side effects from antidepressant treatment as measured by the Toronto Side Effects Scale (TSES)[12].

3.6. Participant timeline

3.6.1. Study Timeline

**TIMEPOINT**	Intake Pre-Study	Day 0	Day 1	Day 7	Day 14	Day 21	Day 28	Day 42
**VISIT NUMBER:**	1	2	3	4	5	6	7	8
**ENROLLMENT:**								
Eligibility screen	x							
Informed consent	x							
Allocation		x						
**INTERVENTIONS:**								
Allocated Treatment		x		x	x	x		
Pregnancy test (If applicable)		x		x	x	x		
**ASSESSMENTS:**								
Baseline data		x						
**Blood tests:** Vitamin B12		x						

Homocysteine and CBC		x		x	x	x		
**Questionnaires:** HAMD, MINI, ATHF-SF	x							
MADRS, GAD-7, QIDS, and TSES		x	x	x	x	x	x	x
Adverse Events		x	x	x	x	x	x	x

Participants will be enrolled in the study for a total of 6 weeks. Participants receive the first allocated treatment on day 0, followed by subsequent treatments on day 7, 14 and 21. Follow-ups will occur by phone according to the schedule above.

3.7. Sample size

Because the anticipated effect size is unknown, a formal sample size calculation cannot be completed. For this pilot study, it is anticipated that forty participants will be recruited. If feasible, the effect size will be used to inform sample size for the full study.

3.8. Recruitment

Recruitment will occur at the Centre for Depression and Suicide Studies at St. Michael's Hospital. The clinic receives approximately 100 referrals per year. An estimated 30% of these referrals would be eligible for inclusion in this trial.

3.9. Allocation

An online random number generator will be used to generate an allocation sequence in random permuted blocks (www.sealedenvelope.com). The allocation list will be sent to a research staff member from Research Pharmacy at the St Michael's Hospital who will prepare the allocation packages. When a participant comes for the study procedure, the study coordinator will call the Research Pharmacy staff to prepare a study package, containing either midazolam or normal saline. Midazolam or normal saline vials will be placed in an obscure paper bag, that will look the same to keep the study coordinator blinded to the content. The interventional physician will open the package once the participant enters the procedure room, therefore minimizing attrition after randomization. The physician will then prepare the appropriate medication for the intravenous administration (normal saline or midazolam) and the corresponding gas for inhalation (nitrous oxide or oxygen). Participants will receive either 1) study group: inhalation of nitrous oxide + intravenous saline, or 2) placebo group: inhalation of oxygen + intravenous midazolam. Though this method is sufficiently robust for our feasibility trial, we will use web-based central randomization center in the planned future multicenter trial.

3.10. Blinding (masking)

3.10.1. Blinding mechanism

As noted above, only the individual administering the intervention will be aware of allocation assignment due to safety. The participant and outcomes assessors will be blinded to minimize bias.

3.10.2. Emergency unblinding

To maintain the quality of the study, emergency unblinding will only occur when knowledge of the intervention is essential for participant care as determined by the Principal Investigator. In the case of emergency unblinding, the timing, reason for doing so, and personnel involved will be recorded in the case report form and blinding will be maintained in as many other study personnel as possible.

Participants may be unblinded to their group allocation before the study closure. Participants who have completed the study (i.e., completed the 6 weeks follow up) and have requested this information will be provided with their group allocation. The study participants are individuals suffering from a treatment-resistant major depression, who are continuously trying different treatment options; therefore, withholding the information on the assigned group for 1 or 2 years until the study closure may be potentially harmful. The risk of unblinding the study personnel will be low since data collection for the participant would be completed.

3.11. Data collection

3.11.1. Trial procedures and evaluations

3.11.1.1. Pre-study Screening:

Eligible participants will be screened at the Centre for Depression and Suicide Studies at St. Michael's Hospital. Participants diagnosed as suffering from a Major Depressive Disorder (MDD) that meets the criteria from Diagnosis and Statistical Manual for Mental Disorders (DSM-5), will be approached to ask to consider participating in the study.

After participants have provided informed consent, they will be ask to complete the Hamilton Depression Rating Scale (HAMD)[13], the Mini International Neuropsychiatric Interview (MINI)[14] for DSM-5, the Antidepressant Treatment History Form – Short Form (ATHF-SF)[15], and will be asked about their medical comorbidities and concomitant medications to confirm eligibility. If a participant is eligible to participate in the study, the participant will be enrolled in the study and the study coordinator will book an appointment for the participant's study intervention.

3.11.1.2. Baseline Data:

After initial screening and informed consent is obtained, the following data will be collected at baseline:

(1) Demographic information such as age, sex, gender, height, and weight.

(2) Detailed data regarding current medications and medical comorbidities, as well as medication history assessed using the Antidepressant Treatment History Form – Short Form (ATHF-SH)[15].

(3) Major depressive disorder severity will be assessed at baseline using

- The Montgomery-Åsberg Depression Rating Scale (MADRS)[11],
- The General Anxiety Disorder 7-item (GAD-7)[16],
- The Quick Inventory of Depressive Symptomatology(QIDS)[17], and
- Toronto Side Effects Scale (TSES)[12]

(4) Medical history i.e., history of bipolar or psychosis, substance use, electrocombulsive treatment, previous treatment with ketamine

(5) Blood sample (13 ml) for measuring CBC, Vitamin B12 and homocysteine levels

(6) For females of childbearing potential, a urine pregnancy test will be completed to confirm that the patient is not pregnant before starting in the study.

3.11.1.3. Follow-up Data:
For each follow-up visit, participants will complete the MADRS, GAD-7, QIDS, and the TSES to assess outcome measurements. Follow-ups that coincide with treatment administrations will be done in-person prior to the intervention. Otherwise follow-ups will occur over the phone. Participants will be asked for a blood sample to test for CBC and homocysteine levels before each study procedure. For females of childbearing potential, a urine pregnancy test will be required to confirm that the patient is not pregnant prior to each study treatment.

3.11.1.4. Retention:
To promote participant retention and complete follow-up, we will provide participants with telephone reminders twenty-four hours before their scheduled appointments.

3.12. Data management

Study data will be collected via case report forms (CRF). The study data will be entered into an encrypted and password protected Microsoft® Access database (Microsoft Corp., Redmond, WA, USA) by designated research personnel. Appropriate range and missing data filters will be used to address data quality. Data accuracy will be assessed by randomly selecting 10% of individuals who will have their data confirmed by a second reviewer.
All records pertaining to the trial will be retained and stored for a period of 25 years as per Health Canada requirements.

3.13. Statistical methods

3.13.1. Outcomes

Based on the primary endpoints, we will determine whether or not this pilot study is:

1. *Feasible*
   All feasibility outcomes are met, no protocol modifications are needed;
2. *Feasible with modification*
   All feasibility outcomes are met or can be met with protocol modifications;
3. *Not feasible*
   Even with protocol modifications, some feasibility outcomes cannot be met.

In terms of statistical analyses for this feasibility study, preliminary statistical analysis will consist of univariate tests to compare clinical and demographic variables of interest. Linear regression will be used to quantify the strength and magnitude of the relationship between the intervention and the primary outcome. Secondary outcomes will be analyzed as continuous measures using the generalized linear regression models. In addition to comparing the responses based on two time points (e.g. baseline and week 4), the changes in scores over the follow-up duration will be investigated using generalized linear mixed models.

3.14. Data monitoring

3.14.1. Formal committee

Given that this is a feasibility trial with a small sample size, no Data Safety Monitoring Committee will be formed.

3.14.2. Interim analysis
There will be no planned interim analysis for this feasibility trial.

3.15. Safety/harms
3.15.1. Safety of the Intervention:
Nitrous oxide is a commonly administered anesthetic with a demonstrated safety profile in the concentrations used for this study. There are case reports of hematologic effects after a single administration however these are considered rare. As there is a potential for nitrous oxide to inactivate vitamin B12, we will measure plasma homocysteine levels and obtain a complete blood count (CBC) before each study treatment.

Addiction and abuse of nitrous oxide has been reported, also physical and psychological dependence may occur during benzodiazepine treatment. The risk is more pronounced in patients on long-term or high-dose treatment and in predisposed patients, such as those with a history of alcoholism, drug abuse or marked psychiatric disorders. In order to minimize the risk of dependence, midazolam and nitrous oxide will be administered at the lowest dose possible and we will exclude patients who may be predisposed.

Body spaces may be prone to changes in volume due to nitrous oxide transfer; we will exclude participants with pneumothorax, pneumocephalus, and recent middle ear, vitreoretinal or bowel obstruction surgeries.

Nitrous oxide crosses the placenta, therefore we have excluded pregnant women from the study. Even if nitrous oxide was not found to influence the initiation or continuation of breastfeeding when used during labor[18], we are excluding participants who are breastfeeding during this feasibility trial because Midazolam (placebo) can be found in the breast milk of lactating women.[19]

The nitrous oxide delivery system (Porter Sentry) will be connected to active scavenging in order to reduce the potential for nitrous oxide pollution affecting research staff and care-team members. The Porter Sentry System is designed to operate on medical nitrous oxide and medical oxygen from cylinders and/or medical gas piping systems. The Porter Sentry System is licenced in Canada

After considering the above mentioned risks of Nitrous Oxide and Midazolam administered by trained anesthesiologists weighed against the severely limited options currently available for treatment resistant depression, the potential benefit that this study may bring to patients is certainly justifiable.

All potential adverse events in this study will be reviewed by the study investigators using the criteria set forth below.

3.15.2. Adverse Event Definitions
Adverse event (AE): An AE is any untoward medical occurrence in a study participant receiving an investigational medicinal product (IMP) and which does not necessarily have a causal relationship with this treatment. An AE can therefore be any unfavorable and unintended

sign (including an abnormal laboratory finding), symptom, or disease temporally associated with the use of an IMP, whether or not related to the IMP.

Adverse drug reaction (ADR): An ADR is any noxious and unintended response to an IMP related to any dose. The phrase 'response to an IMP' means that a causal relationship between the IMP and an AE carries at least a reasonable possibility, i.e., the relationship cannot be ruled out.

3.15.3. Collection of AEs

The condition of the participant will be monitored throughout the study. At each visit, whether scheduled or unscheduled, AEs will be elicited using the Toronto Side Effects Scale. In addition, the Investigator will check the participant records for any documented event.

Any AE or ADR which occurs during the study will be noted in detail on the appropriate pages of the CRF. If the participant reports several signs or symptoms representing a single syndrome or diagnosis, the diagnosis should be recorded in the CRF. The Investigator will grade the severity of all AEs or ADRs (mild, moderate, or severe), the seriousness (non-serious or serious), and the likelihood that they were related to the IMP (causality). The investigator will be responsible for assessing the expectedness of each ADR (expected or unexpected).

Diseases, signs and symptoms, and/or laboratory abnormalities already present before the first administration of IMP will not be considered AEs unless an exacerbation in intensity or frequency (worsening) occurs.

The Investigator will provide detailed information about any abnormalities and about the nature of and reasons for any action taken as well as any other observations or comments that may be useful for the interpretation and understanding of an AE or ADR.

3.15.4. Severity of AEs

The intensity/severity of AEs will be graded as follows:

Mild: an AE, usually transient, which causes discomfort but does not interfere with the participant's routine activities

Moderate: an AE which is sufficiently discomforting to interfere with the participant's routine activities

Severe: an AE which is incapacitating and prevents the pursuit of the participant's routine activities

The grading of an AE is up to the medical judgement of the Investigator and will be decided on a case-by-case basis.

3.15.5. Causality of AEs

All AEs will be assessed by a blinded Investigator as to whether they can be explained by the participant's underlying condition. If they are not explainable, then the Investigator will make a determination of the relationship of the AE with the IMP as follows:

Probable: reports including good reasons and sufficient documentation to assume a causal relationship, in the sense of plausible, conceivable, likely, but not necessarily highly probable. A reaction that follows a reasonable temporal sequence from administration of the IMP; or that follows a known or expected response pattern to the suspected medicine; or that is confirmed by stopping or reducing the dosage of the medicine and that could not reasonably be explained by known characteristics of the participant's clinical state.

Possible: reports containing sufficient information to accept the possibility of a causal relationship, in the sense of not impossible and not unlikely, although the connection is uncertain or doubtful, for example because of missing data or insufficient evidence. A reaction that follows a reasonable temporal sequence from administration of the IMP; that follows a known or expected response pattern to the suspected medicine; but that could readily have been produced by a number of other factors.

Unlikely: reports not following a reasonable temporal sequence from IMP administration. An event which may have been produced by the participant's clinical state or by environmental factors or other therapies administered.

Not related (unrelated): events for which sufficient information exists to conclude that the etiology is unrelated to the IMP.

Unclassified: reports which for one reason or another are not yet assessable, e.g., because of outstanding information (can only be a temporary assessment).

3.15.6. Classification of ADRs by Expectedness

ADRs will be classified by the investigator as either expected or unexpected:

Nitrous Oxide

Expected: an ADR that is listed in the current edition of the Nitrous Oxide Product Monograph (Lexicomp®): (Frequency not defined)

A significant reaction like rash; hives; itching; red, swollen, blistered, or peeling skin; fever; wheezing; tightness in the chest or throat; trouble breathing, swallowing, or talking; unusual hoarseness; or swelling of the mouth, face, lips, tongue, or throat.

Cardiovascular System: Hypotension.

Central Nervous System: Central nervous system stimulation, confusion, dizziness, headache. Prolonged use may produce neurologic dysfunction; patients with vitamin B12 deficiency (pernicious anemia) and those with other nutritional deficiencies (alcoholics) are at increased risk.

Gastrointestinal: Nausea and vomiting. Occurs postoperatively in ~15% of patients.

Respiratory: Apnea

Bone Marrow Suppression: Prolonged use may produce bone marrow suppression; patients with vitamin B12 deficiency (pernicious anemia) and those with other nutritional deficiencies (alcoholics) are at increased risk.

Body Space Volume Expansion: Both compliant (eg, bowel gas, pneumothorax) and poorly compliant (eg, middle ear) body spaces may be prone to changes in volume due to nitrous oxide transfer.

Addictive: May be associated with abuse and/or addiction.

Midazolam

Expected: an ADR that is listed in the Midazolam Product Monograph (MIDAZOLAM INJECTION):

Sedative effects and fluctuations in vital signs were the most frequent reported adverse experiences. The more frequently encountered fluctuations in vital signs included decreased tidal volume and/or decreased respiratory rate and apnea, as well as variations in blood pressure and pulse rate.

Cardiovascular: Increased mean arterial pressure (8%), decreased mean arterial pressure (29.9%), increased pulse rate (29.9%), decreased pulse rate (16.8%)

Respiratory: Increased respiratory rate/tachypnea (36.9%), decreased repiratory rate (25.6%), apnea (1%)

Other adverse reactions occurring at a lower incidence, usually less than 1%, are:

Cardiovascular: Premature ventricular contractions, bigeminy, vasovagal episode, bradycardia, tachycardia and nodal rhythm.

Respiratory: Laryngospasm, bronchospasm, dyspnea, shallow respiration, hyperventilation, and wheezing.

Central Nervous System/Neuromuscular: Nervousness, restlessness, anxiety, argumentativeness, aggression, insomnia, nightmares; deep sedation, prolonged sedation, oversedation, disorientation, slurred speech, emergence delirium, agitation during emergence, prolonged emergence from anesthesia, dreaming during emergence; dysphoria, euphoria, anterograde amnesia, lightheadedness, feeling faint; tremors, muscle contractions, twitches and abnormal spontaneous muscular activity, tonic/clonic movements, athetoid movements; ataxia.

Gastrointestinal: Acid taste, excessive salivation and retching.

Special Senses: Blurred vision, diplopia, nystagmus, visual disturbance, difficulty focusing eyes, pinpoint pupils, cyclic movement of eyelids, ears blocked and loss of balance.

Dermatological: Erythema, rash, pruritus and hives.

Hypersensitivity: Allergic reactions, including anaphylactic shock.

Miscellaneous: Muscle stiffness, toothache, yawning, cold feeling when drug injected and cool sensation in arm during infusion.

Unexpected: an ADR that is not listed in the current edition of the Nitrous Oxide Product Monograph or the Midazolam Product Monograph that differs because of greater severity or greater specificity.

3.15.7. Outcome of AEs

The outcome of all reported AEs has to be documented as follows:

1. Recovered, resolved
2. Recovering, resolving
3. Not recovered, not resolved (by Study Completion visit)
4. Recovered, resolved with sequelae
5. Fatal
6. Unknown

3.15.8. Procedures to manage Adverse Events

Action(s) taken

AEs requiring action or therapy must be treated with recognized standards of medical care to protect the health and well-being of the participant. Appropriate resuscitation equipment and medicines must be available to ensure the best possible treatment in an emergency situation. The action taken by the Investigator must be documented:

1. General actions taken in the event of an AE
   a) None
   b) Medication (other than IMP) or other (e.g., physical) therapy started
   c) Test performed
   d) Other (to be specified)
1. IMP-related actions taken in the event of an AE
   a) None
   b) Product withdrawn
   c) Dose reduced
   d) Dose increased

The Investigator will follow up on each AE until it has resolved or until the medical condition of the participant has stabilized. Any relevant follow-up information will be reported to the Principal Investigator (Sponsor).

3.15.9. Serious Adverse Events (SAEs)

A **serious AE (SAE)** is any untoward medical occurrence that at any dose:

- results in death,
- is life-threatening (see below),
- requires hospitalization or prolongation of existing hospitalization,
- results in persistent or significant disability/incapacity,
- is another important medical event.

NOTE: The term ‘life-threatening’ refers to an event in which the participant was, in the view of the reporting Investigator, at immediate risk of death at the time of the event; it does not refer to an event which may hypothetically have caused death had it been more severe.

In deciding whether an AE/ADR is serious, medical judgement will be exercised. Thus, important AEs/ADRs that are not immediately life-threatening or do not result in death or hospitalization but may jeopardize the participant or may require intervention to prevent one of the other outcomes listed in the definitions above should also be considered serious.

3.15.10. SAE Reporting Timelines

Reporting to REB: The study investigator will report to the REB all SAEs that are unexpected AND there is a reasonable possibility that the SAE is related to the research study, within seven calendar days of the study team becoming aware of the event. All fatal or life-threatening SAEs that are unexpected AND there is a reasonable possibility that the SAE is related to the research will be reported within 3 days. Follow-up reports of the SAE will be submitted to the REB whenever new relevant information regarding the SAE becomes available until the resolution of the SAE.

Reporting to Health Canada: The study investigator as the study sponsor is required to inform Health Canada, in an expedited manner, of any serious unexpected adverse drug reaction, in respect of the study drug:
a) Where it is neither fatal nor life-threatening, within 15 days after becoming aware of the information;
b) Where it is fatal or life-threatening, within 7 days after becoming aware of the information. Within 8 days after having initially informed Health Canada of the fatal or life-threatening ADR, submit as complete a report as possible. Follow-up reports of fatal or life-threatening reactions must include an assessment of the importance and implication of the findings, including relevant previous experience with the same or similar drugs.

The following safety relevant information should be reported as an AE or, if the reaction fulfils one of the criteria for seriousness, as an SAE:

a) Drug overdose

An overdose is a deliberate or inadvertent administration of a treatment at a dose higher than specified in the protocol and higher than the known therapeutic dose that is of clinical relevance. The reaction must be clearly identified as an overdose.

b) Drug interaction

A drug interaction is a situation in which a substance or medicinal product affects the activity of an IMP, i.e., increases or decreases its effects, or produces an effect that none of the products would exhibit on its own. The reaction must be clearly identified as a drug interaction.

c) Medication error

A medication error involves the inadvertent administration or unintended use of a medicinal product which may be caused by the naming, presentation of pharmaceutical form/packaging, or instructions for use/labelling. The reaction must be clearly identified as a medication error.

3.16. Auditing

For this feasibility trial there will be no pre-specified independent audit. Representatives of the St. Michael's Hospital including St. Michael's Hospital Research Ethics Board may look at the study records and at personal health information to verify that the information collected for the study is correct and to make sure the study is following proper laws and guidelines.

4. ETHICS AND DISSEMINATION

4.1. Research ethics approval

This study will be conducted in accordance with the ethical principles laid down in the Declaration of Helsinki, the protocol, Good Clinical Practice guidelines, and applicable regulatory requirements. Submission for Health Canada approval will be made by the Sponsor.

Full written informed consent will be obtained prior to conducting any study activities. The study will be reviewed and approved by the Research Ethics Board before any study related procedures commence.

4.2. Protocol amendments

Before any changes to the study are implemented, beside those to eliminate immediate hazard to study participants, an amendment to the study will be reviewed and approved by the Research Ethics Board and Health Canada as applicable.

4.3. Informed consent process

Prior to completing the survey, these participants will be approached by the study coordinator, who in turn will provide them information about the purpose of the study.The participants will be provided with sufficient time to read the informed consent form, and ask any questions before deciding to participate. Participants who consent to participate in the study will then be asked to sign an informed consent form. These recruitment and consenting procedures will be carried before the psychiatrists and anesthetists provide clinical care of the study participants.

4.4. Declaration of interests

The principal investigators of this trial have no financial or competing interests to declare.

4.5. Ancillary and post-trial care

No additional provisions will be made for post-trial care and routine clinical care will be provided by the participant's primary physician. If the participant suffers harm as a result of a study intervention, appropriate follow-up care will be provided as indicated.

4.6. Dissemination policy

4.6.1. Trial results

The results of this trial will be published in a peer-reviewed journal and presented at scientific conferences/meetings.

4.6.2. Authorship

To be eligible for authorship on any resultant publications, all potential contributors must fulfill all criteria as set forth by the International Committee of Medical Journal Editors.

5. STUDY ADMINISTRATION

5.1. Key contacts

Study Principal Investigator
Karim S. Ladha, MD MSc
St. Michael's Hospital and the University of Toronto
[redacted]

Study Co-Principal Investigator
Venkat Bhat, MD MSc
St. Michael's Hospital and the University of Toronto
[redacted]

5.2. Funders

St. Michael's Hospital Medical Services Association Innovation Fund

5.3. Roles and responsibilities

5.3.1 Sponsor and funder

The study funder has no role in study design, collection, management, analysis and interpretation of data; writing of the report; and the decision to submit the report for publication.

6. REFERENCES

1. Collaborators G 2017 D and II and P, James SL, Abate D, et al. Global, regional, and national incidence, prevalence, and years lived with disability for 354 diseases and injuries for 195 countries and territories, 1990-2017: a systematic analysis for the Global Burden of Disease Study 2017. *Lancet Lond Engl*. 2018;392(10159):1789-1858. doi:10.1016/s0140-6736(18)32279-7

2. Rizvi SJ, Grima E, Tan M, et al. Treatment-Resistant Depression in Primary Care across Canada. *Can J Psychiatry*. 2014;59(7):349-357. doi:10.1177/070674371405900702

3. Vutskits L. General Anesthetics to Treat Major Depressive Disorder. *Anesth Analg*. 2018;126(1):208-216. doi:10.1213/ane.0000000000002594

4. Vutskits L, Xie Z. Lasting impact of general anaesthesia on the brain: mechanisms and relevance. *Nat Rev Neurosci*. 2016;17(11):705-717. doi:10.1038/nrn.2016.128

5. Naughton M, Clarke G, O'Leary OF, Cryan JF, Dinan TG. A review of ketamine in affective disorders: current evidence of clinical efficacy, limitations of use and pre-clinical evidence on proposed mechanisms of action. *J Affect Disorders*. 2013;156:24-35. doi:10.1016/j.jad.2013.11.014

6. Trullas R, Skolnick P. Functional antagonists at the NMDA receptor complex exhibit antidepressant actions. *Eur J Pharmacol*. 1990;185(1):1-10. doi: 10.1016/0014-2999(90)90204-j.

7. Berman RM, Cappiello A, Anand A, Oren DA, Heninger GR, Charney DS, Krystal JH. Antidepressant effects of ketamine in depressed patients. *Biol Psychiatry*. 2000;47(4):351-4. doi: 10.1016/s0006-3223(99)00230-9.

8. Nagele P, Duma A, Kopec M, et al. Nitrous Oxide for Treatment-Resistant Major Depression: A Proof-of-Concept Trial. *Biol Psychiatry*. 2015;78(1):10-18. doi:10.1016/j.biopsych.2014.11.016

9. Nagele P, Myles P. Nitrous Oxide for the Treatment of Major Depressive Disorder. *ClinicalTrials.gov National Library of Medicine (U.S.)*. Identifier NCT03869736. (2019, March 11 -). Retrieved May 14, 2021 from: https://clinicaltrials.gov/ct2/show/NCT03869736

10. Guimarães MC, Guimarães TM, Hallak JE, Abrão J, Machado-de-Sousa JP. Nitrous oxide as an adjunctive therapy in major depressive disorder: a randomized controlled double-blind pilot trial. *Braz J Psychiatry*. 2021;S1516-44462021005005201. doi:10.1590/1516-4446-2020-1543.

11. Montgomery SA, Åsberg M. A New Depression Scale Designed to be Sensitive to Change. *Br J Psychiatry*. 1979;134(4):382-389. doi:10.1192/bjp.134.4.382

12. Vanderkooy JD, Kennedy SH, Bagby RM. Antidepressant Side Effects in Depression Patients Treated in a Naturalistic Setting: A Study of Bupropion, Moclobemide, Paroxetine,

Sertraline, and Venlafaxine. *Can J Psychiatry*. 2002;47(2):174-180. doi:10.1177/070674370204700208

13. Hamilton M. (1960). A rating scale for depression. *J Neurol Neurosurg Psychiatry*. 1960;23(1),56. doi: 10.1136/jnnp.23.1.56.

14. Sheehan DV, Lecrubier Y, Harnett-Sheehan K, et al. The Mini International Neuropsychiatric Interview (M.I.N.I.): The Development and Validation of a Structured Diagnostic Psychiatric Interview. *J Clin Psychiatry*. 1998;59(suppl 20): 22-33.

15. Sackeim HA, Aaronson ST, Bunker MT, et al. The assessment of resistance to antidepressant treatment: Rationale for the Antidepressant Treatment History Form: Short Form (ATHF-SF). *J Psychiatr Res*. 2019;113;125–136. doi:10.1016/j.jpsychires.2019.03.021

16. Spitzer RL, Kroenke K, Williams JBW, Löwe B. A Brief Measure for Assessing Generalized Anxiety Disorder: The GAD-7. *Arch Intern Med*. 2006;166(10):1092. doi:10.1001/archinte.166.10.1092

17. Rush AJ, Trivedi MH, Ibrahim HM, et al. The 16-Item quick inventory of depressive symptomatology (QIDS), clinician rating (QIDS-C), and self-report (QIDS-SR): a psychometric evaluation in patients with chronic major depression. *Biol Psychiatry*. 2003;54(5):573-583. doi:10.1016/s0006-3223(02)01866-8

18. Zanardo V, Volpe F, Parotto M, et al. Nitrous oxide labor analgesia and pain relief memory in breastfeeding women. *J Matern Fetal Neonatal Med*. 2018;31(24);3243-3248. doi:10.1080/14767058.2017.1368077

19. Nitsun M, Szokol JW, Saleh HJ, et al. Pharmacokinetics of midazolam, propofol, and fentanyl transfer to human breast milk. *Clin Pharmacol Ther*. 2006;79(6);549-557. doi:10.1016/j.clpt.2006.02.010
